# Photosynthetic Reactions: From Molecules to Function, and from Simple Models to Complex Systems

**DOI:** 10.3390/ijms231911180

**Published:** 2022-09-23

**Authors:** Katarzyna B. Gieczewska, Beata Myśliwa-Kurdziel, Joanna Grzyb

**Affiliations:** 1 Department of Plant Anatomy and Cytology, Faculty of Biology, University of Warsaw, I. Miecznikowa 1, 02-096 Warsaw, Poland; 2 Department of Plant Physiology and Biochemistry, Faculty of Biochemistry, Biophysics and Biotechnology, Jagiellonian University, Gronostajowa 7, 30-387 Krakow, Poland; 3 Department of Biophysics, Faculty of Biotechnology, University of Wrocław, F. Joliot-Curie Street 14a, 50-383 Wrocław, Poland

Photosynthesis is the basic process for life on Earth—and the one that has changed life history most drastically. When you consult a textbook, the definition of photosynthesis is the assimilation of CO_2_, powered by light, usually accompanied by O_2_ release. Simple? In fact, the mechanism behind the phenomenon is tremendous! The process has already been intensively investigated for centuries. Yes, its official discovery by Jan Ingenhousz is dated to 1779, but some partial observations were made even earlier. We already know and understand a lot. However, there are even more pieces missing from the final picture. Photosynthesis is being investigated on several levels of organisation. The most obvious are the whole plant or the cell levels. However, we can then look into the organisation and functioning of photosynthetic organelles, selected parts of the photosynthetic membrane, and pigment–protein complexes. To study the function of the latter, we often need to build a model system, allowing us to investigate individual factors isolated from the whole net of dependencies inside the plant organism.

The enzyme which still draws attention is Ribulose-1,5-bisphosphate carboxylase/oxygenase (RuBisCo), which catalyses the actual incorporation of carbon dioxide molecules. However, the enzyme is far from perfect, and actually oxidises its substrate in the presence of elevated oxygen concentrations. As such, not only is CO_2_ assimilation missing, but the substrate molecule is also used inefficiently. The reasons for these two routes are somewhere in the enzyme mechanism, but we do not know where exactly. This is because CO_2_ fixation includes several transient steps which are hard to catch in experiments. They can, however, be assessed in silico, as shown by Fedunov & Sokolov [1]. Based on density theory and quantum chemical modelling, they confirmed equal probability of the formation of 2,3-enediol and 1,2-enol due to ribulose-1,5-bisphosphate enolisation. Broadly understood in silico research is also helping researchers to develop a deeper understanding of the enzyme functioning in vivo. This is the case in the study presented by Rydzy et al. [2]. The authors worked on a model, built in silico, of RuBisCo from a dinoflagellate representative, *Symbiodinium* sp.; they used it to identify a unique loop in the protein sequence, probably involved in an interaction with a chaperone-like protein during the enzyme assembly or with a protein functioning as an activity modulator. An in silico investigation was the only possibility, as the RuBisCo of *Symbiodinium* is not active in heterologous systems. Therefore, achieving its crystal structure has not been possible so far. The findings from in silico investigation, both at the level of the reaction mechanism and of the function of protein fragments, can be used in further experimental designs for biotechnological improvements to RuBisCo.

The CO_2_ fixation yield is also regulated on different levels to RuBisCo, for example, using mechanisms of CO_2_ concentration existing in specialised cells or cell compartments. The availability of ATP and NADPH, which are necessary for the sequential steps of the Calvin cycle, are additional regulatory factors. This chemical force is the result of light-driven reactions; it occurs due to light absorption by photosynthetic antennae and follows processes occurring in the membrane or in membrane-built pigment–protein complexes. This means there needs to be a regulatory mechanism correlating light-dependent photosynthetic reactions and CO_2_ assimilation. However, is it the same in all types of plants? This question was raised by Shimikawa & Miyake [3], who examined, in detail, gas exchange, chlorophyll fluorescence, electrochromic shift, and near-infrared absorbance in intact leaves of maize (an NADP-malic enzyme C4 subtype species) in comparison with mustard (a C3 plant). A significant difference was shown in the capacity for photorespiration. The optimisation of photosynthetic reactions to light by C4 plants under varied light conditions is well summarised by Wasilewska-Dębowska et al. [4]. After a detailed introduction of the types of C4 photosynthesis, a comprehensive description of adaptive mechanisms—including the efficient dissipation of excess energy in photosynthetic antennae, balanced energy distribution between photosystems, and the regulation of electron transport methods—are reviewed. The adaptive/acclimatization plasticity of C4 plants is compared with C3 plants.

Light quality is crucial for photosynthesis. This is not only the intensity but also the wavelength range. The latter is defined by the absorption spectrum of chlorophylls and other auxiliary pigments, which may be found within the photosynthetic apparatus. The variety of these antenna pigments is especially found in cyanobacteria and algae. An example is phycobilisomes, which broaden light absorption to a green spectral range. An interesting example of cyanobacteria is *Gloeobacter violaceus*, which is unique due to not having thylakoids. Its photosynthetic machinery is located directly in the plasma membrane, which leads to proton gradient (ΔpH) creation across this structure. 

Additionally, special rhodopsin molecules are built in the same membrane, and may help with ΔpH formation. Kula-Maximenko et al. investigated the growth of *Gleobacter* under blue–red light supplemented with a far-red, ultraviolet A, and green light. Interestingly, they found that the light composition influenced not only the overall yield of biomass production, but also the shape of the cells, as well as inducing changes in particular components of the light-dependent reaction chain [5]. At most, this influenced the content of the auxiliary antennae. Such adaptation, which is broadly known as environmental plasticity, is not only a feature of single-cell organisms. Additionally, higher plants, with the model plant *Arabidopsis* among them, may adapt to the environment. This, of course, has not only light as a factor, but also other biotic and abiotic components of an ecosystem. This may also lead to genetic variation among a species. For *Arabidopsis*, which is studied worldwide, this means that the wild-type of one laboratory does not necessarily have the same characteristics (including broadly understood photosynthetic characteristics) as the wild-type used in another laboratory. Wójtowicz & Gieczewska performed a detailed characterisation and comparison of the photosynthesis performance and spectral properties of the photosynthetic apparatus in the *Arabidopsis thaliana* accessions Col-0, Col-1, Col-2, Col-8, Ler-0, and Ws-2, commonly used as background lines [6]. They found discrepancies in the arrangement of the pigment–protein complexes, the photosynthetic complex ratios, and the lateral mobility of the thylakoid membranes. This basically means that studying photosynthesis in a model plant is still not a simple task, and selecting an accession suitable for specific research on the photosynthetic process is essential for optimising experiments.

Nevertheless, photosynthetic membranes do not only function due to the presence of pigment–protein complexes. There are also lipids. Amongst them, there are isoprenoid quinones and chromanols. The most important role of these molecules is protection. They act as antioxidants against reactive oxygen species generated as a result of electron transfer chain leakage and other possible oxidative damage. To learn more about this issue, the reader should refer to the review by Nowicka et al. [7]. 

Finally, we want to drive readers’ attention toward the work by Velikova et al. [8]. They investigated changes in the leaf morphology, chloroplast ultrastructure, and photosynthetic activity of pea plants treated with single-walled carbon nanotubes. This paper is interesting due to its examination of the effects of nanomaterials, such as carbon nanotubes, on photosynthesis. Nanomaterials, increasingly used in industry and in everyday products, are becoming pollutants. Due to their nano-size, they may penetrate into plants’ bodies, and—as proven by Velikova et al.—also their cells. As nanomaterials’ properties differ from their bulk material of origin, it is necessary to not base our knowledge on approximation, but to look into the details of how they influence such crucial process as photosynthesis. Nanomaterials, however, offer a lot of possibilities, especially when combined with structures of natural origin, as with photosynthetic components. These are the biosensors, elements of artificial light-harvesting systems, and nanocarriers for substances that are beneficial for plant growth. We believe this is the future of photosynthesis exploration and application.

**Footnotes:** For the paper of Rydzy M. et al. titled “Insights into the Structure of Rubisco from Dinoflagellates—In Silico Studies” Int. J. Mol. Sci. 2021, 22(16), 8524, the correct funding information is: grant number 2019/33/N/NZ3/01168, with funding from the National Science Centre, Poland.

## Data Availability

Not applicable.
